# Clinicians perspectives towards the application of shared decision making in tertiary CVD care including the multidisciplinary heart team

**DOI:** 10.1016/j.ijcha.2025.101657

**Published:** 2025-03-26

**Authors:** Mirjam M. Garvelink, Tom Oirbans, Lea M. Dijksman, Paul B. van der Nat, Dennis van Veghel, Daniela N. Schulz, Marcel G.W. Dijkgraaf, Lucas V.A. Boersma

**Affiliations:** a IQHealthcare, RadboudUMC, Nijmegen, the Netherlands & Department of Value Based Healthcare, St. Antonius Hospital, Nieuwegein, the Netherlands; b Department of Value Based Healthcare, St. Antonius Hospital, Nieuwegein & Department of Cardiology, Nieuwegein, the Netherlands; c Department of Value Based Healthcare, St. Antonius Hospital, Nieuwegein, the Netherlands; d Department of Value Based Healthcare, St. Antonius Hospital, Nieuwegein & IQHealthcare, RadboudUMC, Nijmegen, the Netherlands; e Department of Cardiology and Cardiothoracic Surgery, Catharina Hospital Eindhoven, the Netherlands; f Department of Epidemiology and Data Science, Amsterdam UMC, University of Amsterdam, the Netherlands; g Department of Cardiology, St. Antonius Hospital Nieuwegein, the Netherlands

**Keywords:** Shared decision-making, Cardiovascular disease, Barriers, Tertiary center

## Abstract

**Background:**

Shared decision-making (SDM), is a sine qua non in healthcare. Yet, it has been difficult to implement SDM in routine practice for patients with cardiovascular disease(CVD). To improve this, we aimed to determine HCPs perspectives on their SDM behavior in CVD context and influencing factors, and with special focus on multidisciplinary heartteams.

**Methods:**

Cross-sectional survey between March-July 2022 with cardiologists, cardiothoracic surgeons, medical residents, and nurse practitioners within two of the largest tertiary cardiac Centers in the Netherlands. Descriptive statistics were used for quantitative data; open-ended questions were thematically analyzed.

**Results:**

72 participants completed the survey. Respondents indicated to know “very well” what SDM entailed (70 %) and had positive attitudes towards SDM (90 %). Participants used SDM in daily practice (SDMQDoc = 73/100), but indicated that more SDM could be performed (67 %). In self-reported definitions of SDM, explaining the consequences of treatment (step 2) and discussing patients’ preferences (step 3) were most frequently mentioned. Barriers for SDM were patient and process characteristics: e.g. lack of time (70 %), understaffing (35 %). The heartteam was seen as potential facilitator, but its current role and process were seen as barrier for SDM. Facilitators for SDM were managerial support (16 %), decision aids (28 %), and SDM-training (13 %).

**Conclusion:**

HCPs reported high knowledge and application of SDM, but overlooked some steps in the SDM-process. A multifaceted intervention is needed focusing on awareness, enhancing communication skills and system level support. Specific improvements were identified to improve SDM for patients discussed by the multidisciplinary heartteam.

## 1 Introduction

Value-based health care (VBHC) [1] is increasingly being embraced to improve value of care by focusing on quality instead of quantity of provided care [2]*.* Aspects of VBHC are monitoring and benchmarking outcomes of care with patients and professionals, to identify and implement improvements for *groups of patients* [3]. Another important aspect of VBHC is to communicate outcomes with *individual patients*, to support them in Shared Decision-Making (SDM) about which treatment option best supports their desired quality of life (QoL) [4].

Shared decision making is the process in which patients are involved in treatment decision-making, in order to provide the most appropriate care, adapted to the patient’s situation, goals, preferences and what matters in their lives. Advantages of SDM are for example reductions in decisional conflict and better informed decisions [5], [6]. SDM is particularly relevant when multiple treatments are available, and when patients' values are relevant to determine the best decision [7]. For example, when options may have serious side effects that potentially affect patients’ quality of life, such as interventions for cardiovascular diseases (CVD).

Most patients with (complex) CVD are referred from peripheral hospitals to tertiary care centers, to determine and provide appropriate treatment options. These patients are then discussed in the heartteam [8], consisting primarily of a cardiologist and cardiothoracic surgeon, who advise on the most appropriate treatment methods based on clinical and anatomical characteristics of the patient.

Especially in CVD care, the adoption of SDM has been slow [9], [10] despite availability of cardiac care-specific tools to support SDM [11], [12] growing focus on education and training [13] and recommendations for SDM in guidelines [14], [15]. The role of the heartteam has come forward as a potential barrier for SDM in CVD care [16], yet exact barriers and facilitators for SDM in CVD care remain unclear. To improve the implementation of SDM in CVD care, insight is essential in the culture (including professional skills, attitudes), context (“standard“ CVD care, role of the heartteam) and needs as perceived by HCPs. We aimed to determine attitudes, behaviors, intentions and needs of HCPs towards SDM, in two specialized cardiac centers with multidisciplinary heartteams.

## 2 Methods

### 2.1 Study design

Cross sectional survey between March and July 2022.

### 2.2 Participants

Cardiologists, cardiothoracic surgeons, medical residents, and nurse practitioners (HCPs) within two of the largest tertiary cardiac Centers in the Netherlands (n = 86 at St Antonius Hospital in Nieuwegein and n = 36 at Catharina Hospital in Eindhoven).

### 2.3 Survey

**Fig. 1:**
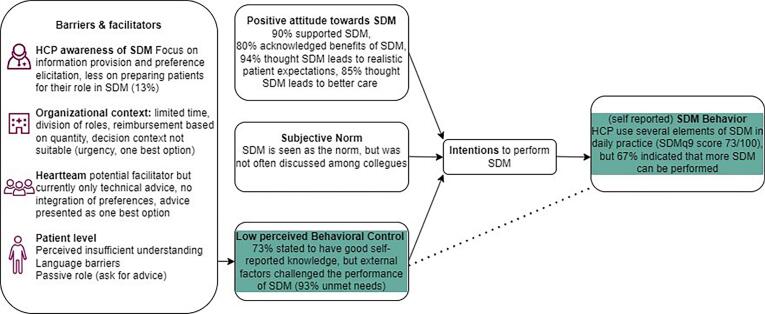
Schematic visualization of the results as per the theory of planned behavior (Azjen(17)).

The survey (Appendix 1), based on the theory of planned behavior (TPB) [17], consisted of 20 self-developed questions or statements (with possibility to add open comments), 3 open ended questions, one reformulated version of a validated questionnaire (SDMQdoc scale). The TPB has been used often to study HCPs behaviors, and specifically SDM behaviors [18], [19]. This theory states that the adoption of behavior can be predicted by someone’s intentions to engage in that behavior, which are influenced by beliefs about attitudes, subjective norms and perceived behavioral control (Fig. 1).

By measuring components of behavioral intentions, insight can be gained in the current situation and needs to optimize SDM behavior. To increase practical relevance of results, we assessed relevant factors specific to SDM in tertiary CVD care such as the heartteam. The survey was available online or on paper.

### 2.4 Data analysis

Descriptive statistics were used. Continuous data were reported as mean +/- SD and categorical data as frequencies (percentage). We present the data as one group as we did not find significant differences in the responses from the two cardiac centers (data not shown).

A total score for the Shared Decision Making Questionnaire-Doc Version (SDM-Q-Doc) was calculated and transformed into a 0–100 score (higher score indicates more SDM). Further, the SDM-Q-Doc items were mapped into the four-step SDM model of Stiggelbout [20] to identify improvement potential in the SDM process:

1) inform the patient that a decision needs to be made and that the patient's opinion is important (items 1–3)
2) explain options,pros and cons (items 4–5)
3) discuss patient’s preferences to support deliberation of options(items 6–7)
4) make or defer a decision, discuss a possible follow-up (items 8–9)

Responses to statements that were measured on a scale with six answer categories, were recoded into (strongly) agree (1,2) and (strongly)disagree (4,5). The options “neutral” and “I do not know” remained the same.

Answers to open-ended questions and explanatory comments were categorized by two independent reviewers (TO and MG). Disagreements between reviewers were resolved through discussion.

For self-reported definitions of SDM and reasons for (not) performing SDM, we used existing frameworks from the literature [7], [20] to categorize the open answers.

### 2.5 Ethics

The United Medical Research Ethics Committees concluded that this study (**R&D/Z22.010)** did not fall under the Medical Research Involving Human Subjects Act (WMO). No ethics approval was needed. This study conforms to the ethical guidelines of the 1975 Declaration of Helsinki and was conducted in accordance with local laws and regulations. Written informed consent was obtained.

## 3 Results

### 3.1 Sociodemographic characteristics and work settings

Of the 123 HCPs that were invited, 77 HCPs responded (63 %); 72 HCPs (59 %) completed the survey, and 5 HCPs (4 %) opted out. See Table 1 for participants’ baseline characteristics.

**Table 1:** Baseline characteristics.

**Characteristics**	**N = 72 (%)**
**Cardiac center**	
St Antonius Hospital	49 (68)
Catharina hospital	23 (32)

**Sex**	
Male	45 (63)
Female	27 (37)

**Age group**	
25 to 34 years	18 (25)
35 to 44 years	20 (28)
45 to 54 years	19 (26)
55 to 64 years	15 (21)

**Work experience current cardiac center**	
< 1 years	4 (6)
1 to 4 years	13 (18)
5 to 9 years	15 (21)
10 to 14 years	14 (19)
15 to 19 years	9 (12)
≥ 20 years	17 (24)

**Profession**	
Medical specialist	37 (51)
Medical resident	16 (23)
Nurse practitioner	19 (26)
HCP has a role in the multidisciplinary heartteam*, yes	33 (89)

**Specialism**	
Cardiology	49 (68)
Cardiothoracic surgery	22 (31)

**Sub specialism (if cardiology)**	
General Cardiology	12 (25)
Imaging	8 (16)
Electrophysiology	10 (20)
Interventional Cardiology	10 (20)
Other	9 (19)

**Which percentage of your patient contacts involves…**	
Emergency settings	14 (17)
Frail patients	37 (25)

* Only for specialists (n = 37).

### 3.2 Attitude

**Fig. 2:**
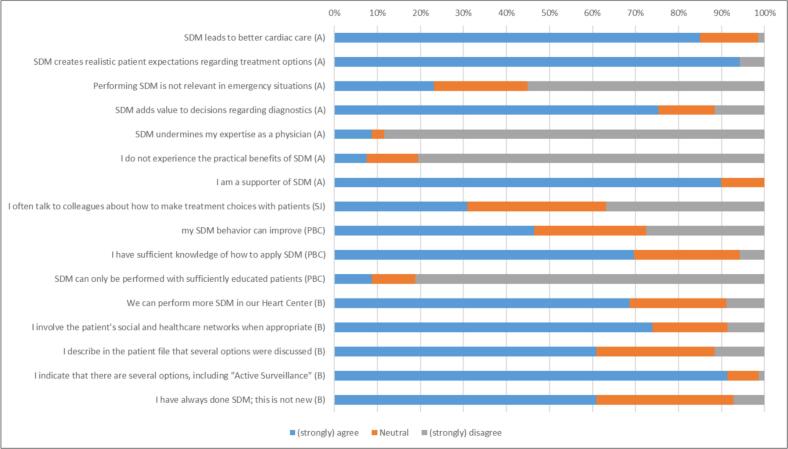
Responses to statements about attitude (A), subjective norms (SJ), perceived behavioral control (PBC), and behavior (B) (N_max_ = 69).

All statements were in favor of SDM (Fig. 2). sixty-two respondents supported SDM in general (90 %). Most participants disagreed that SDM can only be done with patients who are sufficiently educated to discuss options with their HCP (n = 56, 81 %). Almost all participants agreed that SDM leads to realistic patient expectations (n = 65, 94 %) and better cardiac care (n = 57, 85 %).

### 3.3 Subjective norm

Two-thirds of the participants stated that more SDM can be done within the Heart Center than currently done (n = 46, 67 %). Frequently discussing SDM with colleagues is not common practice (n = 21, 30 %). (Fig. 2) One respondent added:*” I cannot judge how things are going with my colleagues but I hope this “SDM strategy” is already being applied.”*.

### 3.4 Perceived behavioral control

Seventy-three percent (n = 51) of the participants self-rated their knowledge of SDM as “*fair to (very) good*”; 24 % (n = 17) indicated “*some familiarity*” with the concept of SDM. In self-reported definitions of SDM, respondents most often mentioned step 2 (n = 41, 64 %) and step 3 (n = 39, 61 %). Step 1 (n = 8, 13 %) and step 4 (n = 13, 20 %) were least mentioned. The majority of HCPs indicated to have unmet needs to perform SDM (n = 67, 93 %) related to 1) Process redesign; 2) Resources and training; 3 Attitude/culture (Table 3).

### 3.5 Intentions

Respondents gave positive ratings for the necessity of SDM (n = 63, 97 %), patient desirability (n = 59, 91 %), effective use of resources (n = 59, 91 %), confidence in skills (n = 60, 92 %) and importance despite clinical preference (n = 57, 88 %). In situations where the stakes are low, the majority of HCPs (n = 52, 80 %) are comfortable providing care that is not aligned with their clinical recommendation. Less so when the stakes are high (35 %).

### 3.6 Current behavior

#### 3.6.1 Activities related to SDM

Sixty-one percent of HCPs indicated that they have always been doing SDM (n = 42). The majority reported to perform all relevant activities related to SDM (Fig. 2).

**Fig. 3:**
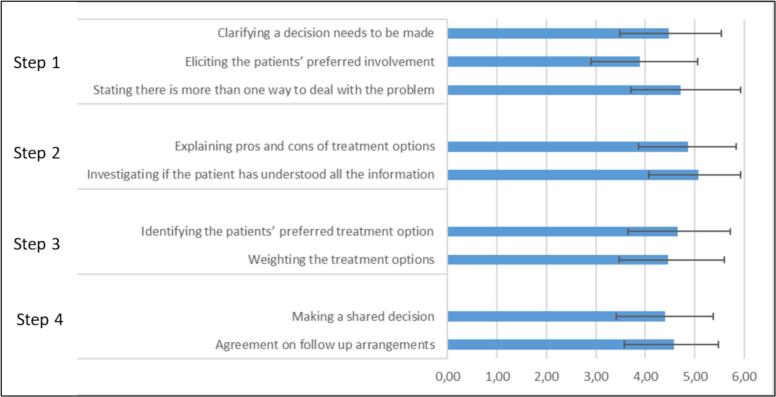
**SDMQ-doc categorized into the four Shared Decision-making steps.** Mean score ± SD of the SDM-Q-DOC items per SDM step. Scores indicate the extent to which respondents perceive themselves to conduct SDM elements. 0 = not at all, 6 = totally applicable. Step 1: inform the patient that a decision needs to be made; Step 2: explain the options and their pros and cons; Step 3: discuss patient’s preferences; Step 4: make or defer a decision and to discuss follow-up.

The median SDM-Q-Doc score was 73 (IQR = 60–82). Lower scores were reported on items related to SDM-step 1 and 4 (Fig. 3). One respondent added: “*The doctor is responsible for the indication. The patient is responsible for the consent. [..] making shared decisions is very straightforward in this context*.”.

#### 3.6.2 Possible situations for SDM

Situations suitable for SDM occur either weekly- (n = 25, 36 %) or daily (n = 41, 59 %). Most participants indicated that they perform SDM “almost always” (n = 34, 49 %) or “about half the time” (n = 30, 44 %) when the situation is suitable. The most frequently reported factors for not performing SDM were related to “Clinical uncertainty/equipoise”; “patient (dis)ability to engage in decision-making”; “limited time”; “Ethical and legal requirements”; and “effects of SDM” (Table 2). Respondents added the difficulty to know whether patients understand the information, and possible language barriers.

**Table 2:** Reasons for (not) performing SDM (number of times reported) as per HCPs.

**Category**	**Reasons for performing SDM**	**Reasons for not performing SDM**
Clinical uncertainty/equipoise	Multiple options (7x)	One best option (6x)
	High impact of decision (7x)	Low impact of the decision (4x)
	Uncertainty of outcomes (3x)	

Patient ability to engage in decision-making	Patient is receptive (5x)	Patient unconscious (4x)
	Patient commitment needed to carry out the decision (8x)	Patient indicates HCP should decide (7x)
		Patient request for therapy in conflict with clinician's judgement (1x)
		Patient has insufficient understanding (17x)

Time	Long time frame to decide (4x)	Limited timeframe (9x)
		Immediate life-saving measures needed (16x)

Ethical and legal requirements	SDM is legal requirement (1x)	Options restricted by legal/institutional policies (2x)
	Every decision (13x)	Not my role in the process (4x)

Effect of SDM	Improved decision-making (19x)	
	Increased patient satisfaction (5x)

**Table 3:** Needs in performing SDM.

*Domain*	*Factors mentioned*	Exemplary Responses:
Process redesign	Lack of time (n = 50, 70 %)Understaffing (n = 25, 35 %)Logistical organization (processes) (n = 11, 16 %)allocation of tasks and responsibilities (n = 10, 14 %)improving infrastructure/facilities” (n = 8, 11 %).	“*For a follow-up patient in the outpatient clinic, you have 10 min to discuss the results of investigations and simultaneously go over all treatment options with their pros and cons, consider the patient's preferences, give the patient time to ask questions, and make a decision. This takes much more time than the allotted 10 min. (r12, hospital A)* “. Another respondent offered a possible solution in his response “*production is prioritized, which creates time pressure. If this pressure is reduced, it will benefit quality care, allow more attention to the patient, and provide time to discuss shared decision-making*.” (R10, Hospital B)

Resources and training.	Easily accessible and integrated decision aids to prepare patients for decision-making outside of the consultation (n = 20, 28 %)Education (n = 18, 25 %) and training in performing SDM (n = 9, 13 %)Budget (n = 4, 6 %)	“*Decision aids would help patients to educate themselves independently, thereby saving time in the hospital”*. “*availability of brochure materials for all treatments at all locations*”.

Attitude/culture	A change in attitude for both staff and patients, in which they listen to each other with respect(n = 20, 28 %)a more lively SDM mindset (n = 13, 18 %).	“*The culture among (cardio)surgeons really needs to change so that trainees do NOT adopt this mindset and continue to focus on the human aspect and the patient, rather than just production and surgery alone*”.

### 3.7 The role of the heartteam in SDM

The majority of the participating medical specialists served on the heartteam (n = 33/39, 85 %). Over a third of the participants indicated that the heartteam (almost) always has a role in determining the treatment strategy (n = 24, 35 %). Most respondents (n = 50, 78 %) found that the heartteam *could, in theory*, be a facilitator for SDM. Currently, many respondents found that the heartteam's advice is not based on patient preferences (n = 35, 53 %); and 42 % thought the heartteam was a barrier for SDM. Thirty-two respondents (46 %) indicated that they, themselves, can perform more SDM. Three assumptions were made with regard to the role for the heart team:

**The heartteam provides technical advice, but the treatment choice is up to the treating HCP and patient.** n = 20, 51 %. Exemplary Responses: “*The Heartteam decision/advice can be discussed with patients later in the context of SDM*”
**The heartteam has a responsibility in the SDM process, but the ability to integrate patient preferences depends on input from the referring HCP.** n = 10, 26 %. Exemplary response: “*SDM does not start within the Heartteam but with the referring HCP*.” Another added: “ *The assessment by the heartteam takes place based on the information from file, then a decision is made, and this is discussed with the patient in the clinic. Patients should be seen more often before the decision is made.”*
**The heartteam advice is perceived as a binding advice.** n = 9, 23 %. Exemplary response: “*The decision of the Heartteam is the starting point for further treatment. It is rare that there is a revision of the decision because the patient wants otherwise.*”

## 4 Discussion

The results of this study show favorable attitudes and behavioral intentions towards SDM. HCPs report to perform SDM on a regular basis but not in *all* suitable situations due to barriers on the level of the patient, clinician and the organization/system level (including the heartteam). These results led us to make three observations:

First, similar to others, [9], [16]^,^ we found a positive attitude towards SDM and widespread willingness to further incorporate SDM in daily practice [21], [22]. Experienced barriers for SDM were for example time constraints, understaffing, roles and allocated tasks, and lack of tools/training [23], [24]. Interestingly, some participants mentioned the quantity-driven reimbursement system as barrier for SDM. This indicates that a more integrated vision on VBHC, could improve implementation of SDM. Moreover, SDM is key in the achievement of sustainable high quality, patient centered care, and increasingly seen as an important part of VBHC [4], [25].

Second, in line with others [24], [26], [27], we found patient-level barriers such as HCPs’ perception that patients do not understand the information, and their preference for the doctor to decide, since “doctors are the experts”. These barriers could be removed by adapting the way the SDM-process is explained to patients (step 1 of an SDM process). This explanation influences patients’ receptiveness for information about options, and openness to explaining their views (on values, preferences) that are of utmost importance for a good SDM process [28]. Our results indicated that a majority of HCPs left out this step in their description of SDM. Also in self-reported SDM behavior (as per the SDMQ9doc), steps 1 and 4 of the SDM process scored lower than other steps. For the full SDM process, multifaceted interventions are needed focusing on each of the four SDM steps, including SDM skills training for HCPs, deliberation tools, and patient preparation and support for their role in decision-making [29].

Third, participants indicated that the Heartteam, could both be a barrier and facilitator for SDM. In its current form, the heartteam seemed no particular facilitator of SDM because the team's advice is not based on patient preferences, yet sometimes presented as only option. As a consequence, HCPs are reluctant to deviate from it [16]. In literature, the best method to ensure MDTs in general are patient centered ánd efficient remains unclear [30]. At the minimum, for SDM to occur, it is important to assess what matters to the patient, before an advice is provided. To ensure the heartteam can facilitate SDM, three elements have been mentioned [16]: 1) input from the treating specialist on patient preferences for the heartteam, 2) mentioning multiple options instead of thé best option in recommendations, and 3) different ways of communicating recommendations to the patient.

### 4.1 Strengths and limitations

Data were collected in two of the largest cardiac centers in the Netherlands. We used validated measures supplemented with self-developed situation-specific statements. Our aim was to inventorize barriers and facilitators for implementation of SDM in our hospitals, not to generalize findings abroad. Given the multiple facets of healthcare logistics in different contexts, the proposed suggestions as to how to improve SDM may not be universal. However, the similarities we found with the literature suggest that our results may be generalizable. A cross sectional survey of HCP in two tertiary cardiac centers has inherent limitations, including possible selection bias and socially desirable answers. We recommend for future research to *observe* the actual SDM process and assess the correlation of SDM adherence with patient outcomes.

## 5 Conclusion and implications

Most elements of the TPB were in favor of SDM, except for perceived behavioral control. Barriers were found on three levels: the patient, the clinician, and the organization/system, including the heartteam. To facilitate SDM in CVD care, better integration in VBHC, and more (patient and HCP) awareness of roles and behaviors in SDM is needed. This can be achieved with tools, training and system level changes. The heartteam can facilitate SDM when patient preferences are central in referral, discussion, formulating recommendations or discussing recommendations with patients. With these results, we can: 1) develop and implement interventions that address the experienced challenges for SDM in CVD care; 2) determine the most relevant outcomes to measure the impact of interventions; 3) increase HCP’s awareness of SDM.

## Data availability

Data is available upon reasonable request with the author.

## Sources of funding

This research was funded by Zorginstituut Nederland/Zonmw Project number: 10070012010005.

## CRediT authorship contribution statement

**Mirjam M. Garvelink:** Writing – review & editing, Writing – original draft, Supervision, Methodology, Formal analysis, Conceptualization. **Tom Oirbans:** Writing – review & editing, Writing – original draft, Project administration, Methodology, Formal analysis, Data curation, Conceptualization. **Lea M. Dijksman:** Writing – review & editing, Supervision, Methodology, Conceptualization. **Paul B. van der Nat:** Writing – review & editing, Supervision, Methodology, Funding acquisition, Conceptualization. **Dennis van Veghel:** Writing – review & editing, Methodology, Data curation, Conceptualization. **Daniela N. Schulz:** Writing – review & editing, Validation, Methodology, Conceptualization. **Marcel G.W. Dijkgraaf:** Writing – review & editing, Supervision, Methodology, Conceptualization. **Lucas V.A. Boersma:** Writing – review & editing, Supervision, Methodology, Funding acquisition, Conceptualization.

## Declaration of competing interest

The authors declare the following financial interests/personal relationships which may be considered as potential competing interests: [Lucas V. A. Boersma, PhD reports financial support was provided by ST. ANTONIUS HOSPITAL. If there are other authors, they declare that they have no known competing financial interests or personal relationships that could have appeared to influence the work reported in this paper].
