# Supplementary material for: Clinicians perspectives towards the application of shared decision making in tertiary CVD care including the multidisciplinary heart team
Source: Int J Cardiol Heart Vasc. 2025 Mar 26;58:101657. doi: 10.1016/j.ijcha.2025.101657 (PMC11985153; doi:10.1016/j.ijcha.2025.101657)
Supplement: Supplementary Data 1 [file mmc1.docx]

**Appendix 1 Survey (in English)**

**Socio-demographic characteristics and work settings**

1. What is your gender?

□ Man

□ Woman

2. What is your age?

□ 24 years or younger

□ 25 to 34 years

□ 35 to 44 years

□ 45 to 54 years

□ 55 to 64 years

□ 65 years or older

3. How long have you been working for St. Antonius Hospital?

□ Less than 1 year

□ Between 1 and 4 years

□ Between 5 and 9 years

□ Between 10 and 14 years

□ Between 15 and 19 years

□ 20 years or longer

4. What is your current position?

□ Medical specialist (partnership)

□ Medical specialist (Spidma, fellow, chef de Clinique)

□ Nurse Practitioner or Physician Assistant

□ Resident (AIOS)

□ Non-specialist doctor (ANIOS)

□ Other, namely_____

5. In which specialty are you working?

□ Cardiology -> go to question 6

□ CTC -> go to question 7

□ Other, namely_____

6. In which sub-specialty are you (primarily) working?

□ General cardiology

□ Imaging

□ Congenital

□ Electrophysiology

□ Interventional cardiology

□ All of the above sub-specialties

□ Other, namely_____

7. In what percentage of your patient contacts is there an emergency setting?

_____% (estimate)

8. In what percentage of your patient contacts is there a fragile or vulnerable patient?

_____% (estimate)

**Knowledge**

9. Do you know what Shared Decision Making (also known as Samen Beslissen) entails?

□ Not at all -> go to question 11.

□ I have heard of it, but do not know the content

□ A little/ only in general terms

□ Fairly to well informed

□ Very well

10. Can you describe in your own words what you understand by Shared Decision Making?

…………………………………………………………………………………………………………………………………………………………

Definition

Shared Decision Making (Samen Beslissen) is the process in which the doctor and the patient (and any relatives) jointly discuss which medical treatment best suits the patient's situation, taking into account all options, pros and cons, patient preferences, and circumstances in decisions about their health. (Definition according to the Federation of Medical Specialists)

11. To what extent do you agree with the following statements? You can use the option "Don't know" if necessary.

|  | Strongly agree | Agree | Neutral | Disagree | Strongly disagree | Don't know |
| --- | --- | --- | --- | --- | --- | --- |
| Shared Decision Making leads to realistic expectations of treatment options for the patient. (A) |  |  |  |  |  |  |
| Applying Shared Decision Making is not relevant in emergency situations within the Heart Center. (A) |  |  |  |  |  |  |
| Shared Decision Making is valuable in decisions regarding diagnostics (ECHO, CAG, MRI, etc.). (A) |  |  |  |  |  |  |
| I have sufficient knowledge about how to apply SDM. (PBC) |  |  |  |  |  |  |
| I believe that Shared Decision Making leads to better heart care. (A) |  |  |  |  |  |  |
| I do not experience any benefits of SDM in practice. (A) |  |  |  |  |  |  |
| SDM can only be done with patients who are sufficiently educated to discuss treatment or screening options with their doctor. (PBC) |  |  |  |  |  |  |
| I think we can do more in heart care regarding SDM. (B) |  |  |  |  |  |  |
| I often discuss with colleagues how to make treatment decisions together with a patient. (SN) |  |  |  |  |  |  |
| I have always practiced Shared Decision Making; this is nothing new. (B) |  |  |  |  |  |  |
| I am a supporter of Shared Decision Making. (A) |  |  |  |  |  |  |
| I think I could apply Shared Decision Making better than I currently do. (B) |  |  |  |  |  |  |
| I think we can do more with Shared Decision Making in our Heart Center. (B) |  |  |  |  |  |  |
| Shared Decision Making undermines my expertise as a doctor/nurse specialist. (A) |  |  |  |  |  |  |
| I document in the file that various options have been discussed. (B) |  |  |  |  |  |  |
| I indicate that there are multiple options, with "Conservative management" also being an option. (B) |  |  |  |  |  |  |
| I involve the patient's social and care network in treatment decisions when necessary. (B) |  |  |  |  |  |  |

Attitude (A), subjective norms (SJ), perceived behavioral control (PBC), and behavior (B)

Do you have anything to add to the above? ………………………………………………………………..

…………………………………………………………………………………………………………………………………………………………

**Application in Practice (part 1 of 2)**

12.How do you generally involve patients in deciding about care/treatment?

□ I make decisions about the treatment myself.

□ I make decisions about the treatment myself, but I strongly take my patient's opinion into account.

□ My patient and I make the decision about the treatment together.

□ I leave the treatment decisions to my patient, but they strongly consider my opinion.

□ I leave the decision about the treatment to my patient alone.

13. How often do situations suitable for Shared Decision Making occur in your daily work?

□ Daily

□ Weekly

□ Occasionally

□ Never -> go to question 15.

□ Other, namely_______

14. In what proportion of situations suitable for Shared Decision Making do you also apply it?

□ Almost always

□ About half the time

□ About a quarter of the time

□ Occasionally

□ Never

□ Other, namely_______

15. What are your reasons for applying Shared Decision Making in these situations?

…………………………………………………………………………………………………………………………………………………………

16. What are your reasons for not applying Shared Decision Making in these situations?

…………………………………………………………………………………………………………………………………………………………

**Role of the Heart Team**

17. How often does the Heart Team play a role in determining the treatment strategy you make with your patient?

□ Almost always

□ About half the time

□ About a quarter of the time

□ Occasionally

□ Never

□ Other, namely_______

(if question 4 -> medical specialist)

18. Are you active in one of the Heart Teams?

□ Yes

□ No

19. To what extent do you agree with the following statements?

|  | Strongly agree | Agree | Neutral | Disagree | Strongly disagree | Don't know |
| --- | --- | --- | --- | --- | --- | --- |
| The advice of the Heart Team is not currently based on patient preferences. |  |  |  |  |  |  |
| Due to the role of the Heart Team, Shared Decision Making is difficult to implement in practice. |  |  |  |  |  |  |
| In theory (apart from practical objections), the Heart Team could serve as a facilitating factor in Shared Decision Making. |  |  |  |  |  |  |

Do you have anything to add to the above?

…………………………………………………………………………………………………………………………………………………………

**Application in Practice (part 2 of 2) – adapted version of the SDMQdoc**

20. To what extent do you pay attention to the following elements of Shared Decision Making?

|  | Not applicable at all | Largely not applicable | Rather not applicable | Rather applicable | Largely applicable | Completely applicable |
| --- | --- | --- | --- | --- | --- | --- |
| I make it clear to my patients that a decision needs to be made. |  |  |  |  |  |  |
| I want to know exactly how my patients want to be involved in making the decision. |  |  |  |  |  |  |
| I tell my patients that there are different treatment options for their complaints. |  |  |  |  |  |  |
| I clearly explain the pros and cons of the treatment options to my patients. |  |  |  |  |  |  |
| I help my patients understand all the information. |  |  |  |  |  |  |
| I ask my patients which treatment option they prefer |  |  |  |  |  |  |
| My patients and I thoroughly weigh the different treatment options together. |  |  |  |  |  |  |
| My patients and I choose a treatment option together. |  |  |  |  |  |  |
| I make an appointment with my patients about the next steps. |  |  |  |  |  |  |

**Willingness to Incorporate (intentions)**

21. Please give your opinion on the following statements:

| When more than one reasonable option exists, informing patients about their treatment options, asking for their preferences, and reaching a joint decision is... | |
| --- | --- |
| □ Unnecessary for patient care or | □ Necessary for patient care |
| □ Not desired by most patients or | □ Desired by most patients |
| □ A poor use of my time with patients or | □ A good use of my time with patients |
| □ A skill I feel unsure about using in practice or | □ A skill I feel confident about using in practice |
| □ Not important if there is a strong medical preference or | □ Important, even if there is a strong medical preference |

22. Please give your opinion on the following statement:

| If a well-informed patient, in a situation where little is at stake, prefers a treatment option that is not in line with my medical recommendation, I would feel... | |
| --- | --- |
| □ Uncomfortable providing this care | □ Comfortable providing this care |

23. Please give your opinion on the following statement:

| If a well-informed patient, in a situation where much is at stake, prefers a treatment option that is not in line with my medical recommendation, I would feel... | |
| --- | --- |
| □ Uncomfortable providing this care | □ Comfortable providing this care |

**Needs and Barriers**

24. What needs to happen within our Heart Center to implement Shared Decision Making in your daily practice in the way you desire? Please check the items that you think apply.

| Topic | Explanation |
| --- | --- |
| □ Reducing time pressure |  |
| □ Staff capacity |  |
| □ Financial factors |  |
| □ Changing logistical organization |  |
| □ Attitudinal change |  |
| □ Making the concept more vibrant |  |
| □ Improved infrastructure/facilities |  |
| □ Tasks and responsibilities |  |
| □ Education |  |
| □ Decision aids |  |
| □ Training |  |
| □ Nothing, the application is already going well |  |
| □ Other |  |

25. Finally, do you have any comments or good ideas that you would like to share with us on this topic?

…………………………………………………………………………………………………………………………………………………………

**End of the questionnaire, thank you very much for completing it**
